# Effect of hydrogen sulfide (H_2_S) on the growth and development of tobacco seedlings in absence of stress

**DOI:** 10.1186/s12870-024-04819-w

**Published:** 2024-03-02

**Authors:** Jingcheng Dai, Dingxin Wen, Hao Li, Jingpeng Yang, Xiongfei Rao, Yong Yang, Jiangke Yang, Chunlei Yang, Jun Yu

**Affiliations:** 1 School of Life Science and Technology, Wuhan Polytechnic University, Wuhan, 430023 China; 2 Tobacco Research Institute of Hubei Province, Wuhan , Hubei, 430030 China; 3 State Key Laboratory of Biocatalysis and Enzyme Engineering, School of Life Science, Hubei University, Wuhan, 430062 China; 4 Pilot Base of Food Microbial Resources Utilization of Hubei Province, College of Life Science and Technology, Wuhan Polytechnic University, Wuhan, 430024 China

**Keywords:** Enzymes activities, Hydrogen sulfide, NaHS, Photosynthetic pigments, Tobacco

## Abstract

**Background:**

Hydrogen sulfide (H_2_S) is a novel signaling molecule involved in the growth and development of plants and their response to stress. However, the involvement of H_2_S in promoting the growth and development of tobacco plants is still unclear.

**Results:**

In this study, we explored the effect of pre-soaking or irrigating the roots of tobacco plants with 0.0, 2.0, 4.0, 6.0, and 8.0 mM of sodium hydrosulfide (NaHS) on endogenous H_2_S production, antioxidant enzymatic and cysteine desulfhydrase activities, seed germination, agronomic traits, photosynthetic pigments contents, and root vigor. The results revealed that exogenous NaHS treatment could significantly promote endogenous H_2_S production by inducing gene expression of *D/L-CD* and the activities of D/L-CD enzymes. Additionally, a significant increase in the agronomic traits and the contents of photosynthetic pigments, and no significant difference in carotenoid content among tobacco plants treated with 0.0 to 8.0 mM of NaHS was observed. Additionally, a significant increase in the germination speed, dry weight, and vigor of tobacco seeds, whereas no significant effect on the percentage of seed germination was observed on NaHS treatment. Furthermore, NaHS treatment could significantly increase the activity of superoxide dismutase (SOD) and peroxidase (POD) enzymes, which reduces damage due to oxidative stress by maintaining reactive oxygen species homeostasis.

**Conclusions:**

These results would aid in enhancing our understanding of the involvement of H_2_S, a novel signaling molecule to promote the growth and development of tobacco plants.

## Background

Initially, hydrogen sulfide (H_2_S) was considered a toxic gas harmful to life and the environment. However, studies conducted in the early twenty-first century revealed the biological functions of H_2_S gas in mammal cells [1]. Endogenous H_2_S is involved in various physiological processes, such as regulating the pathogenesis of multiple mammal diseases, including angiogenesis, atherosclerosis, hypertension, and myocardial infarction [2]. Recently, several studies have determined the role of H_2_S in plants. In fact, a study has shown that H_2_S is a bioactive gas produced by plant cells [3]. On the contrary, to the phytotoxic effect of high H_2_S levels, low H_2_S levels are critically involved in the growth and development of plants, along with their resistance to environmental stress [4, 5]. Several studies have shown that plants could synthesize and consume H_2_S. Corn, cucumber, pumpkin, and soybean leaves produce endogenous H_2_S [6]. Harrington and Smith (1980) identified L-cysteine desulfhydrase (LCD) and D-cysteine desulfhydrase (DCD) in cells of tobacco plants, which catalyzes L-cysteine degradation to generate H_2_S [7]. Furthermore, four cysteine desulfhydrases, such as D-cysteine desulfhydrase 1 (At1g48420), D-cysteine desulfhydrase 2 (At3g26115), L-cysteine desulfhydrase 1 (At5g28030), and L-cysteine desulfhydrase (At3g62130) were identified in *Arabidopsis thaliana* [8–10]. In fact, studies have identified more and more cysteine desulfhydrase homologs in different species, including OsDCD1 and OsLCD2 in rice [11].

Studies have shown that H_2_S could protect plants against stress induced by heavy metals, drought, salinity, heat, and cold [1, 5, 12]. A study has shown that stress due to drought increases cysteine desulfhydrase activity, thus enhancing endogenous H_2_S production in Arabidopsis [13]. Some studies have shown that H_2_S promotes stomatal closure under drought stress [1]. Moreover, H_2_S enhances the tolerance of plants to chromium by increasing the rates of photosynthesis and reducing the uptake of chromium [14]. Studies have shown that under high salinity/alkaline conditions, H_2_S reduces the Na^+^ accumulation, the Na^+^/K^+^ ratio, and K^+^ exocytosis by cells of various plants, such as *Triticum*, *Solanum lycopersicum*, *Fragaria ananassa*, *Oryza sativa*, *Arabidopsis, Spartina alterniflora, Populus popularis*, *Populus euphratica Medicago sativa,* and *Malus hupehensis* [15–17]. Some researchers observed that exogenous Ca^2+^ and CaM, together with H_2_S, could be effective in mitigating the damaging effects of high stress, partly by increasing the activity of LCD enzymes and H_2_S accumulation [18]. Furthermore, under abiotic stress, H_2_S could positively influence seed germination, adventitious rooting, and postharvest senescence [19, 20]. Treatment of wheat seeds with exogenous NaHS could reduce damage due to toxic metal stress [21]. Crosstalk between NO and H_2_S could induce thermotolerance in maize seedlings and promote seedling growth [22]. A study showed that hydrogen peroxide (H_2_O_2_) and H_2_S could enhance the germination of seeds by mobilizing the storage protein in mung beans [23]. However, the mechanism by which H_2_S affects the germination of seeds is still unclear.

Tobacco (*Nicotiana tabacum* L.) is one of the most important economic crops and is extensively cultivated in the southern region of China. The aroma of tobacco is pleasing and it is widely used as an aromatic plant. Globally, China is the largest producer, exporter, and consumer of tobacco and accounts for nearly one-third of the total tobacco consumption every year [24]. However, in pursuit of high yield, chemical fertilizers, and major elements are widely used, and organic fertilizers, as well as trace elements, are sparsely used. This has deteriorated the quality of soil for tobacco cultivation, which has increased the incidences of diseases and reduced the quality of tobacco.

In recent years, H_2_S has received considerable attention because of its emerging roles in the regulation of plant growth and development, such as *Triticum aestivum, Zea mays, Cucumis sativus, Arabidopsis, Fragaria ananassa, Pyrus, Malus hupehensis, Sorghum bicolo, Glycine max, Spinacia oleracea, Oryza sativa, Actinidia chinensis, Malus pumila, Musa nana* and etc. [11, 12, 20, 22, 25–30, 54], while, the genus *Nicotiana*, a member of the family *Solanaceae,* consists of about 60 species worldwide, distributed in South America, North America, and Oceania. Four of these species are widely cultivated in China. There is little research on the effect of H_2_S on the growth and development of the genus *Nicotiana,* which is a very important economic crop. Our research can provide guidance for tobacco cultivation.

We have previously demonstrated that exogenous NaHS inhibited the growth of *Ralstonia solanacearum*, which cause tobacco bacterial wilt [31]. However, the effect of H_2_S on the growth and development of tobacco in the absence of stress is still unclear. Therefore, in this study, we have evaluated the effect of the H_2_S donor NaHS on the endogenous H_2_S signaling, the growth and development of plants, root activity, the germination of seeds, D/L-CD (DCD and LCD) enzyme levels, and the antioxidant enzyme activities in tobacco seedlings in the absence of stress. Our results showed that H_2_S, a novel signaling molecule, could promote the growth and development of tobacco plants. Therefore, these findings not only highlighted the important functions of H_2_S in the growth and development of tobacco plants in the absence of stress, but also paving the way for the application of NaHS in field applications. We hope that the findings presented here will provide farmers and the scientific community with the opportunity to further develop H_2_S-based agriculture.

## Results and Discussion

### NaHS triggers endogenous H_2_S signaling in tobacco in the absence of stress

**Fig. 1:**
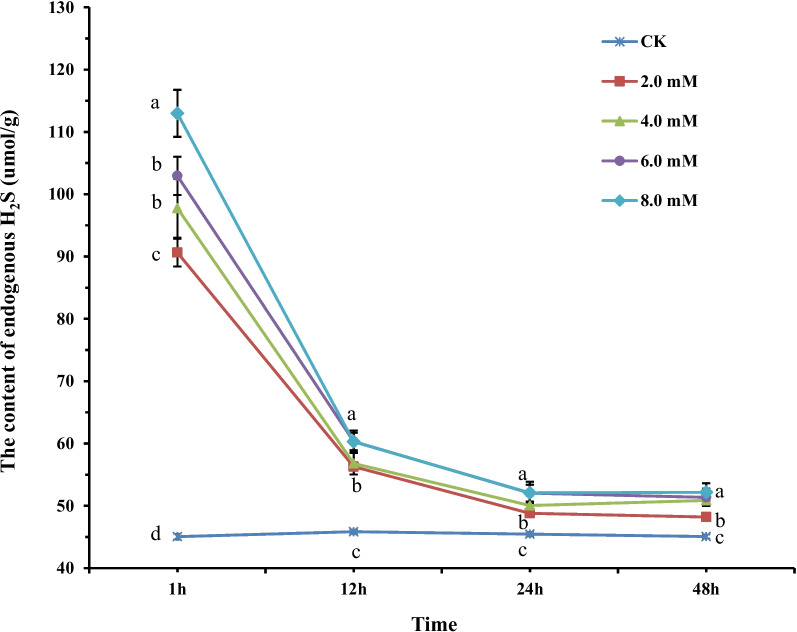
The content of the endogenous H_2_S in tobacco treated with 0.0, 2.0, 4.0, 6.0, and 8.0 mM of NaHS for 1, 12, 24, and 48 h, respectively. The results are the mean of three replicates, and the error bars show the standard deviation (SD). Different letters in lowercase denote significant differences between treatment groups at the 5% level, according to Duncan’s test

**Fig. 2:**
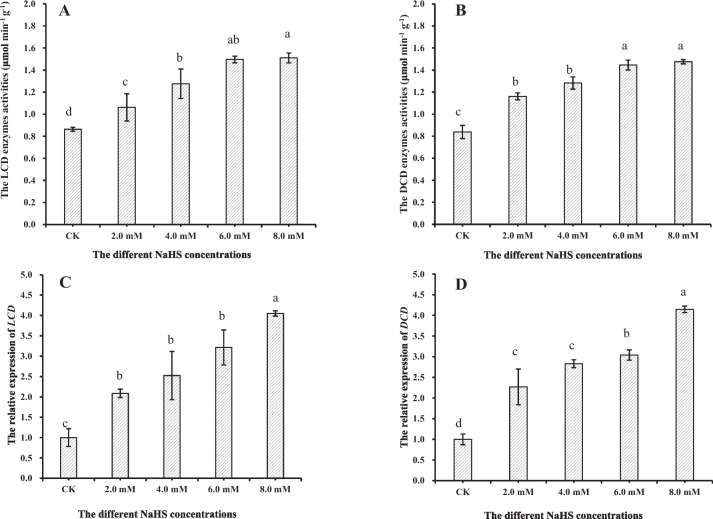
The effect of 0.0, 2.0, 4.0, 6.0, and 8.0 mM of NaHS on the enzyme activities of LCD (**A**) and DCD (**B**), along with *LCD* (**C**) and *DCD* (**D**) expression profiles. The results are the mean of three replicates, and the error bar shows SD. Different letters in lowercase denote significant differences between treatment groups at the 5% level, according to Duncan’s test

First, we determined the efficiency of NaHS treatment in inducing endogenous H_2_S production in tobacco plants. Therefore, we measured the endogenous H_2_S content, *LCD* and *DCD* expression, and their enzyme activities in tobacco exposed to 0.0, 2.0, 4.0, 6.0, and 8.0 mM NaHS. The result showed a significant increase in the content of endogenous H_2_S by 6.96%, 12.87%, 13.97%, and 15.77% in tobacco treated with 0.0, 2.0, 4.0, 6.0, and 8.0 mM NaHS for 1 h, respectively, compared to untreated control (CK) (0.0 mM, Fig. 1). The content of endogenous H_2_S in tobacco stabilized after 12 h. Moreover, no significant difference in the content of endogenous H_2_S was observed among tobacco treated with different concentrations of NaHS after 24 h (Fig. 1). As expected, the results revealed a significant increase in LCD and DCD activities in tobacco treated with NaHS. An increase in enzyme activities was observed in tobacco treated with increasing concentrations of NaHS (Figs. 2A and B). Moreover, a significant increase in *LCD* and *DCD* expression levels was observed in tobacco treated with NaHS. An increase in relative gene expression level was observed in tobacco treated with increasing NaHS concentration (Figs. 2C and D). Our results indicated that H_2_S could induce *LCD* and *DCD* expression for the synthesizing LCD and DCD enzymes. These enzymes could degrade the two-cysteine isomers and produce H_2_S in the absence of other biotic and abiotic stresses in tobacco. Mounting evidence suggests that H_2_S acts as a signaling molecule in plants. Low concentrations of H_2_S play a crucial role in various processes, including the growth and development of plants and their responses to biotic and abiotic stresses [4, 19, 32]. Treating tobacco with exogenous NaHS increases D/L-CD, *O*-acetyl serine(thiol)lyase, cyanoalanine synthase, and carbonic anhydrase enzyme activities, and endogenous cysteine and H_2_S levels under salt-alkaline stress [29, 33]. A study has demonstrated that cold stimulation can enhance D/L-CD enzyme activities and increase endogenous H_2_S production [34]. Similarly, the low exogenous NaHS concentration in tomato and strawberry root cells could increase LCD activity during plug transplant production [27, 35]. Interestingly, our results showed that treating tobacco with exogenous NaHS could increase the D/L-CD enzyme activities and endogenous H_2_S concentration without stress. Recently, a similar study showed that sodium nitroprusside (SNP, as NO donor) could increase *D/L-CD* expression and endogenous H_2_S levels. Moreover, maize seedlings treated with 0.5 mM NaHS enhanced the SNP effects [22], thus implying that H_2_S could directly elevate LCD and DCD enzyme activities in the absence of stress.

### Effect of pre-soaking of tobacco seeds with NaHS on seed germination

**Fig. 3:**
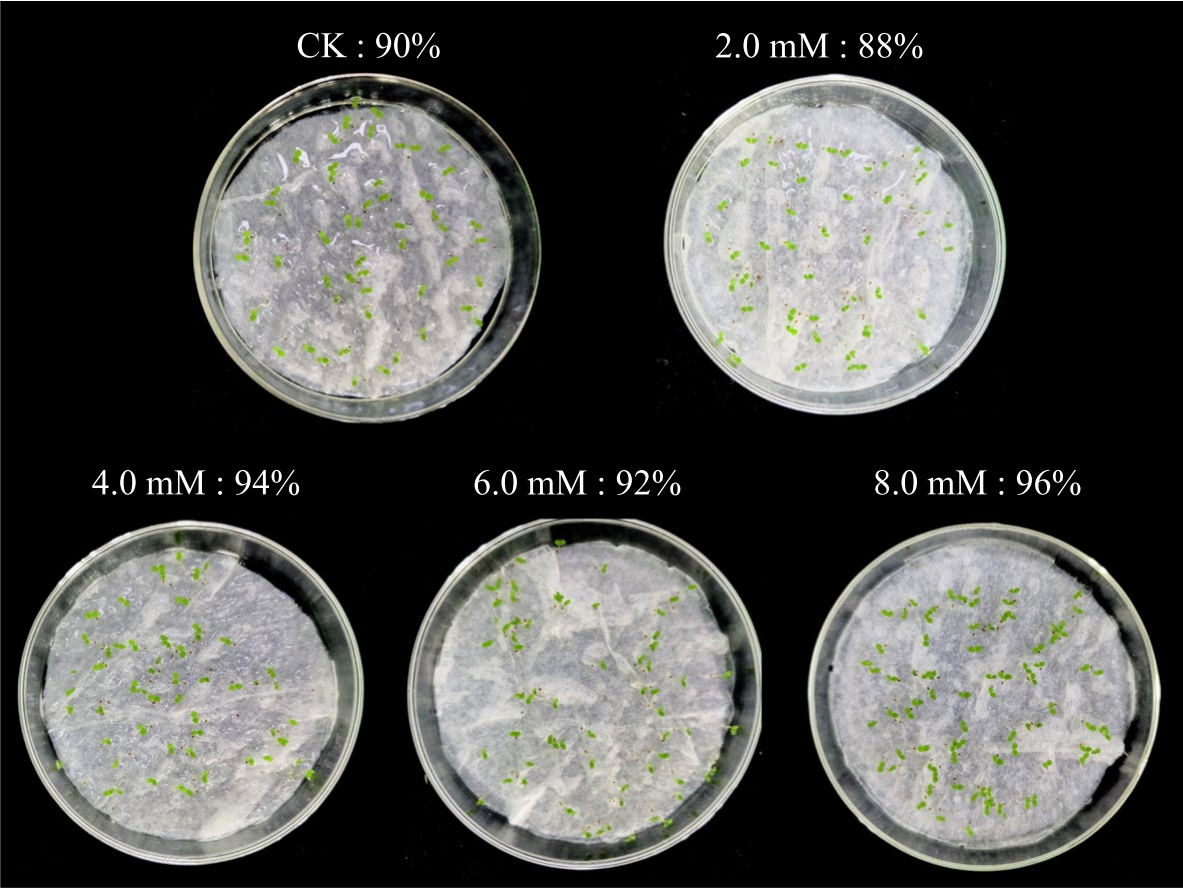
The germination and morphology of tobacco seeds treated with 0.0, 2.0, 4.0, 6.0, and 8.0 mM NaHS for 14 days

Successful germination of seeds is a good starting point in the lifecycle of higher plants. The percentage and speed of seed germination are important since plantlets are very sensitive to biotic and abiotic stresses, and fast germination could benefit seed germination. Recent studies have shown that H_2_S reduces the adverse effects of various factors causing stress on the seed during germination [21, 36, 37]. Zhou et al. showed that H_2_S could induce osmolyte biosynthesis and trigger the antioxidant system at high temperatures to improve the germination process and the growth of maize seedlings [36]. Here, we have determined the effect of H_2_S on tobacco seed germination in the absence of stress. To determine the effect of pre-soaking on the germination and the growth of seedlings in the absence of stress, we pre-soaked tobacco seeds with 0.0, 2.0, 4.0, 6.0, and 8.0 mM NaHS and incubated them in a thermostatic incubator at 28 ℃ for a duration of 14 days. Next, we determined the rate and speed of germination, as well as seed vigor. The results are shown in Fig. 3 and Table 1. The results revealed a significant increase in the speed of germination and vigor of tobacco seeds pre-soaked with 2.0–8.0 mM NaHS compared to the untreated control CK. However, pre-soaking of tobacco seeds with different NaHS concentrations did not significantly affect the percentage of seed germination (Table 1). The speed of germination and seed vigor was highest and increased by 89.83% and 146.87%, respectively, in tobacco seeds pre-soaked with 6.0 mM NaHS compared to CK (Table 1). However, the speed of germination and seed vigor significantly decreased in tobacco seeds pre-soaked with 8.0 mM NaHS (Table 1). These results indicate that H_2_S could improve the germination speed and seed vigor of tobacco seeds and shorten the germination time in the absence of biotic and abiotic stress. A study has shown that H_2_S could improve the seedling size and germination rate and reduce the germination time of corn, bean, pea, and wheat [38]. Li et al. revealed that soaking mung beans with NaHS could increase the endogenous H_2_S and H_2_O_2_ levels in germinating seeds [23]. Meanwhile, H_2_O_2_ treatment increased the rate of *Jatropha curcas* seed germination by stimulating the LCD activity, which triggered H_2_S production [39]. Interestingly, a study showed that endogenous H_2_S signaling could induce resistance to cyanide respiration mediated by the alternative oxidase to enhance seed germination in the absence of biotic and abiotic stress [40].

**Table 1:** Effect of different NaHS concentrations on tobacco seed germination

Treatment	Germination rate (%)	Germination index	Index of Seed Vigor
CK	90 ± 4 ab	16.43 ± 0.29 e	6.57 ± 0.11 e
2.0 mM	88 ± 4 b	20.03 ± 0.36 d	8.81 ± 0.16 d
4.0 mM	94 ± 2 a	21.19 ± 0.35 c	11.02 ± 0.19 c
6.0 mM	94 ± 2 a	31.19 ± 0.19 a	16.22 ± 0.07 a
8.0 mM	96 ± 2 a	26.59 ± 0.14 b	13.22 ± 0.07 b

Different lowercase letters denote statistical differences between treatment groups at the 5% level, according to Duncan’s test

### Effect of irrigating roots with NaHS on the growth and development of tobacco seedlings

**Fig. 4:**
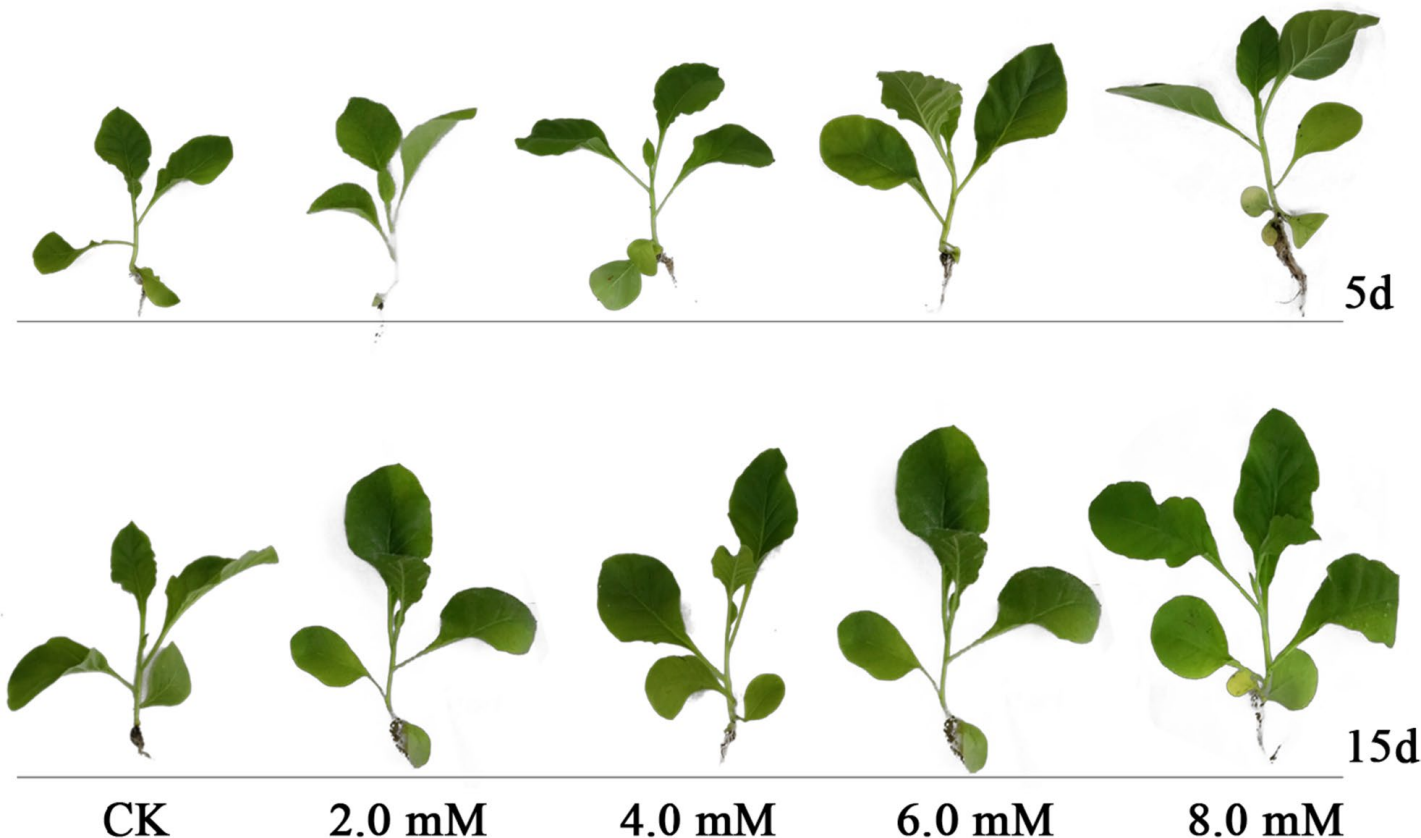
The effect of 0.0, 2.0, 4.0, 6.0, and 8.0 mM of NaHS on tobacco agronomic traits

A study has shown that H_2_S could regulate the growth and development of plants [41]. However, few studies have focused on the effect of exogenous NaHS on promoting the agronomic traits of tobacco in the absence of stress. Therefore, we identified the effect of NaHS on agronomic traits, including the maximum width, length, and area of leaves, stem diameter, and the height of tobacco irrigated with different NaHS concentrations for 15 days. The results showed an increase in the values of tobacco agronomic traits, including the maximum width, length, and area of leaves, stem diameter, and height of tobacco with an increasing NaHS concentration (Fig. 4). The maximum width, length, and area of leaves, stem diameter, and height of tobacco plant treated with 8.0 mM NaHS were 34.13 cm, 23.15 cm, 501.25 cm^2^, 0.91 cm and 38.89 cm, respectively. These agronomic traits significantly increased by 51%, 144%, 187%, 42%, and 44% compared to CK (Table 2). These results indicate that exogenous NaHS treatment could enhance the growth of tobacco plants in the absence of stress. Similar results were observed in three macrophytes, *Potamogeton crispus* *,* *Myriophyllum spicatum,* and *Elodea nuttallii,* exposed to low NaHS concentration. The results showed an increase in the growth of these plants without exhibiting oxidative stress [30]. Moreover, studies have shown a significant improvement in the growth of plants and alleviated the oxidative damage in *Brassica napus* and *Glycine max* plants exposed to low NaHS concentration (0.05–3 mM) under aluminum and drought stress, respectively [42, 43]. However, our result showed that root irrigation with 2.0 mM NaHS had no significant effect on the percentage of seed germination and no increase in maximum leaf width and length. In comparison to other plants, tobacco requires relatively higher concentrations of H_2_S to promote seed germination and seedling growth.

**Table 2:** Effects of different NaHS concentrations on the agronomic traits of tobacco

Treatment	Plant height (cm)	Stem diameter (cm)	Maximum leaf width (cm)	Maximum leaf length (cm)	Leaf area (cm^2^)
CK	26.97 ± 0.87 d	0.64 ± 0.23 d	22.63 ± 1.49 c	9.48 ± 2.35 b	174.36 ± 23.16 e
2.0 mM	31.09 ± 1.79 c	0.70 ± 0.19 c	24.84 ± 1.47 c	12.14 ± 0.84 b	198.38 ± 17.36 d
4.0 mM	34.20 ± 1.47 b	0.86 ± 0.29 b	28.23 ± 2.36 ab	12.59 ± 0.52 b	348.92 ± 20.14 c
6.0 mM	38.77 ± 1.89 a	0.89 ± 0.25 b	33.07 ± 1.65 a	22.75 ± 1.40 a	477.46 ± 14.27 b
8.0 mM	38.89 ± 2.26 a	0.91 ± 0.13 a	34.13 ± 1.21 a	23.15 ± 1.87 a	501.25 ± 22.45 a

Different lowercase letters denote statistical differences between treatment groups at the 5% level, according to Duncan’s test

**Fig. 5:**
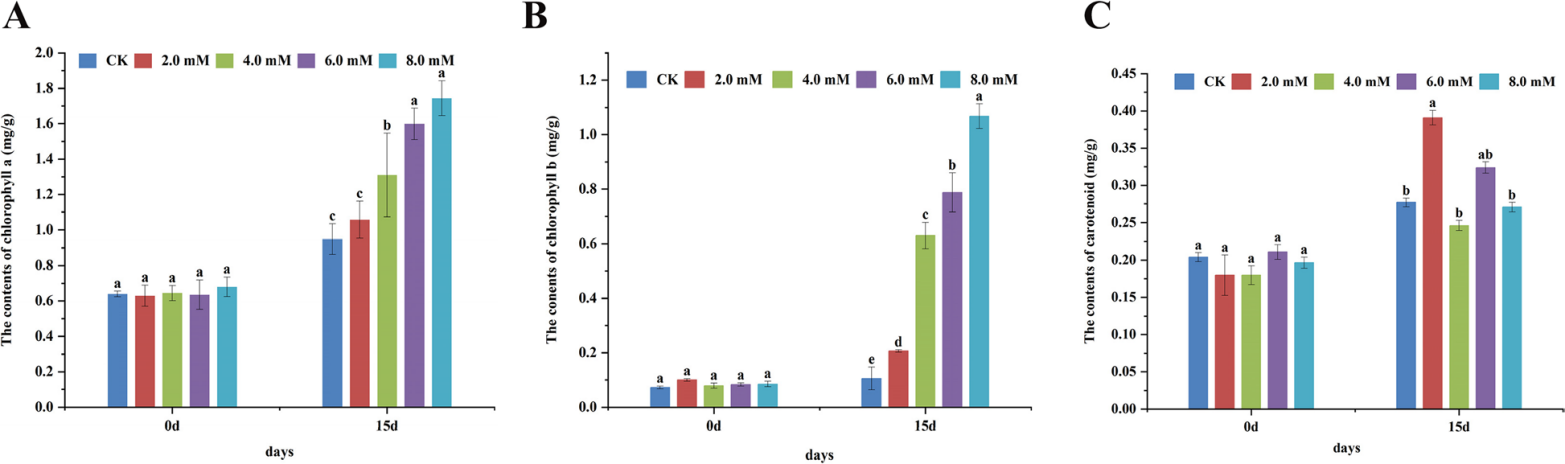
The effect of 0.0, 2.0, 4.0, 6.0, and 8.0 mM of NaHS on Chl *a*, Chl *b*, and carotenoid contents. **A** The contents of Chl *a* in the extracted fresh leaves treated with NaHS on 0 day and 15 days. **B** The contents of Chl *b* in the extracted fresh leaves treated with NaHS on 0 day and 15 days. **C** The contents of carotenoid in the extracted fresh leaves treated with NaHS on 0 day and 15 days. Different letters in lowercase denote significant differences between treatment groups at the 5% level, according to Duncan’s test

Meanwhile, to determine the effect of H_2_S on the development of tobacco leaves and Chl *a*, Chl *b*, and carotenoid contents, we treated fresh leaves with different NaHS concentrations for 15 days and performed a spectrophotometric analysis. No significant difference in terms of Chl *a*, Chl *b*, and carotenoid contents was observed among untreated tobacco plants (Fig. 5). Interestingly, an increase in Chl *a* and Chl *b* contents was observed with increasing NaHS concentrations, compared to CK. However, no significant difference in carotenoid content was observed in tobacco plants treated with NaHS for 15 days (Fig. 5). The result showed that Chl *a* and Chl *b* content in tobacco leaves treated with 8.0 mM NaHS were 1.74 mg/g and 1.07 mg/g, which significantly increased by 83% and 872%, respectively, compared to CK (Fig. 5). These results indicate that H_2_S could increase Chl *a* and Chl *b* content and had no significant effect on carotenoid content in tobacco leaves. Studies have shown that H_2_S could increase the rate of photosynthesis by increasing chlorophyll content in higher-order plants [26, 30, 44] and lower-order plants, such as algae [45]. Thus suggesting that H_2_S could improve the rate of photosynthesis at an early stage of plant evolution to improve the survival of plants. Moreover, endogenous H_2_S production induced by red, blue, or white light in foxtail millet seedlings could play a role downstream of the plant light signal [28]. Furthermore, H_2_S could regulate *LCD* expression and modify the phosphorylation of LCD proteins via two light regulation mechanisms [28]. However, the role of H_2_S in photosynthesis, photomorphogenesis, and light signal transduction is still unclear.

**Fig. 6:**
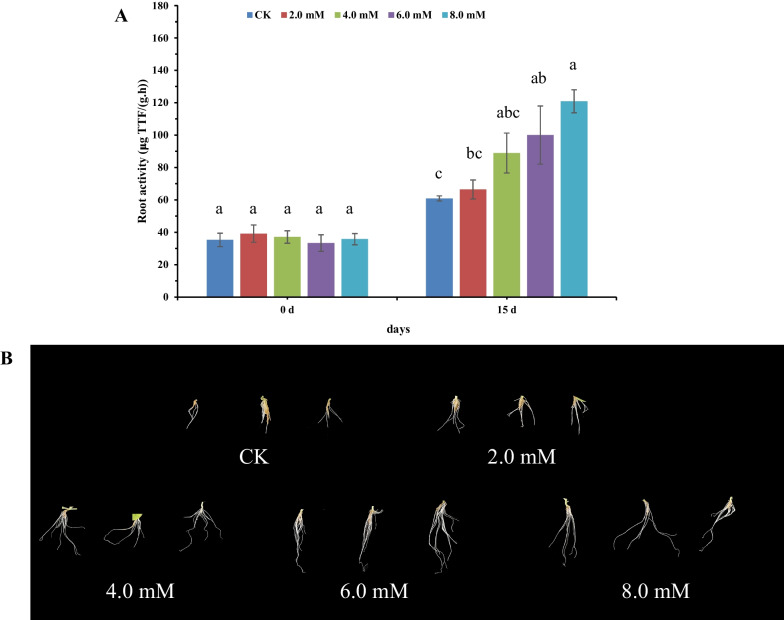
The effects of 0.0, 2.0, 4.0, 6.0, and 8.0 mM NaHS on the activity of tobacco roots after 15 days. Bars represent the mean ± SE. Different letters in lowercase denote significant differences between treatment groups at the 5% level, according to Duncan’s test

Besides, the growth and development of tobacco leaves and the growth and activities of the roots were determined in tobacco treated with different NaHS concentrations for 15 days. The results showed an increase in the dry and wet weight of the root of tobacco exposed to increasing NaHS concentrations. The maximum dry and wet weight of the root of tobacco treated with 8.0 mM NaHS treatment was 0.39 g and 6.02 g. Moreover, the maximum dry and wet weight of the root was the highest, which significantly increased by 144% and 94%, respectively, compared to CK (Table 3). Compared to CK, an increase in root activities was observed with the increasing NaHS concentrations. Moreover, the root activities of tobacco treated with 8.0 mM NaHS treatment were the highest, which significantly increased by 87.83%, respectively, compared to CK (Fig. 6A). Meanwhile, our result also showed that treatment with 6.0 mM and 8.0 mM could significantly promote the primordium initiation and lateral root emergence, compared to CK (Fig. 6B). Some studies have shown that the low NaHS concentration could promote LCD activities in the root cells of tomatoes and strawberry seedlings [27, 35, 46]. This indicates that a low concentration of endogenous H_2_S could directly promote the growth and development of roots. Moreover, high NaHS concentrations (> 2 mM) could inhibit root elongation via the mechanism of suppressing the transport of Auxin in Arabidopsis [1]. Notably, our results showed that high concentrations of exogenous NaHS, i.e., 8.0 mM, could promote root growth activities in tobacco (Fig. 6). This implies that tobacco root cells exhibited tolerance to high concentrations of exogenous NaHS and induced the production of endogenous H_2_S at lower concentrations (115 umol/g), the downstream component of the auxin signaling pathway for triggering the root formation.

**Table 3:** Effects of different NaHS concentrations on the dry and wet weight of tobacco roots

Treatment	Root wet weight (g/6 plants)	Root dry weight (g/6 plants)
CK	3.10 ± 0.18 c	0.16 ± 0.02 b
2.0 mM	3.66 ± 0.15 c	0.19 ± 0.04 b
4.0 mM	4.32 ± 0.06 b	0.20 ± 0.04 b
6.0 mM	5.58 ± 0.10 a	0.37 ± 0.01 a
8.0 mM	6.02 ± 0.43 a	0.39 ± 0.02 a

Different letters with lower indicate significant differences in treatment groups at the 5% level based on Duncan’s test

### Effect of root-irrigation with NaHS on the activities of enzymatic antioxidants

**Fig. 7:**
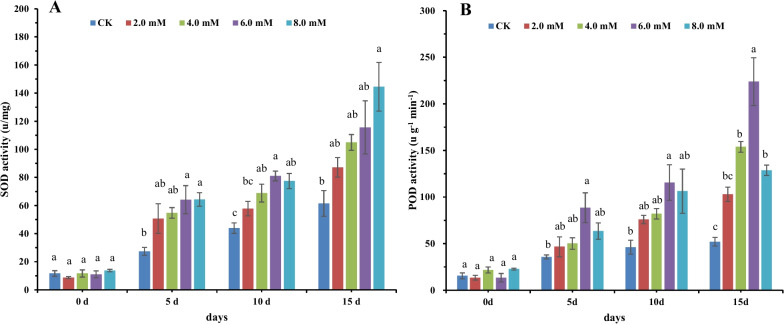
The effects of 0.0, 2.0, 4.0, 6.0, and 8.0 mM of NaHS on the SOD (**A**) and POD (**B**) enzymatic activities in tobacco plants treated for 0, 5, 10, and 15 days. Bars represent the mean ± SE. Different letters in lowercase denote significant differences between treatment groups at the 5% level, according to Duncan’s test

Our results demonstrated that the exogenous application of NaHS could promote endogenous H_2_S production (Fig. 1), which could increase the resistance of plants to various abiotic stresses, thereby promoting plant survival [12, 47]. Continuous exposure to abiotic stress could cause H_2_O_2_ and ROS accumulation, which leads to lipid peroxidation, protein oxidation, and cellular damage [48, 49]. SOD and POD are important enzymes that play a crucial role in eliminating free oxygen radicals and influencing the resistance to stress in plants [1, 34]. To demonstrate the effect of irrigation of tobacco roots with NaHS in promoting the SOD and POD enzymatic activities in the absence of stress, we irrigated tobacco roots with 0.0, 2.0, 4.0, 6.0, and 8.0 mM of NaHS for 5, 10, and 15 days. The results showed no significant differences in the SOD and POD enzymatic activities among untreated tobacco plants. However, an increase in the SOD and POD enzymatic activities was observed with increasing concentrations of exogenous NaHS compared to CK (Fig. 7). Interestingly, no significant difference in SOD and POD enzymatic activities was observed among tobacco treated with 6.0 mM and 8.0 mM NaHS (Fig. 7). Moreover, the results revealed a significant decrease in POD enzymatic activity in tobacco treated with 8.0 mM NaHS, thus, indicating that 6.0 mM of exogenous NaHS is the most suitable for the SOD and POD enzymatic activities in tobacco. Several studies have shown that H_2_S could alleviate oxidative stress by enhancing the gene expression and activities of some antioxidant enzymes, such as SOD, catalase, POD, glutathione reductase, ascorbate peroxidase in plants [16, 34, 50, 51]. These genes could be involved in the post-translational modification mediated by H_2_S. Studies have shown that H_2_S facilitates antioxidant enzymes to eliminate ROS and induces H_2_O_2_ production by increasing NADPH oxidase activity [50, 52, 53]. This regulates H_2_S and ROS levels in plant cells. ROS accumulation causes oxidative stress. Exogenous NaHS could induce endogenous H_2_S production, which could reduce ROS levels by enhancing the antioxidant enzyme activities.

As described above, it was supposed that exogenous application of NaHS could induce gene expression of *D/L-CD* and the activities of D/L-CD enzymes to catalyse L-cysteine degradation to produce endogenous H_2_S, which would subsequently facilitate seed germination by stimulating protease activity and total free amino acid production. However, the NaHS was not effective in breaking the dormancy of the seeds because the germination of the tobacco seeds might be inhibited by abscisic acid (ABA) [12]. Actually, the mechanism for the effect of H_2_S on seed germination is still unclear. Besides, H_2_S might also affect the growth and development of tobacco seedlings with or without biotic and abiotic stress through multiple cross-talk of H_2_S, NO and ROS [54]. In plant cells, the actions, functions and mechanisms of H_2_S, and NO, and H_2_O_2_ are inseparable, and the crosstalk between NO and H_2_S could control the level of ROS by triggering ROS-scavenging system [22]. Meanwhile, H_2_S represents a feedback pathway to enhance signaling cascades by inducing the accumulation of some signaling substances, in particular NO, ABA and Ca^2+^, to maintain cellular redox homeostasis, exchange metal ion transport, alter gene expression and enzyme activities [55]. Multiple types of research need to be done to explore the mechanism of the crosstalk between H_2_S and other signal messengers. At present, it is poorly understood whether H_2_S is involved in photosynthesis, photomorphogenesis or light signaling. Given the importance of photosynthesis in a broad sense, it is promising to continue to investigate the function of H_2_S. Furthermore, H_2_S regulated gene expression not only as a substrate for Cys biosynthesis, and some genes appeared to act on H_2_S signaling independently of Cys [56]. Therefore, plant hormone crosstalk, DNA repair, protein post-translational modification (PTMs), metabolite synthesis and mRNA transcription are all potentially responsive to H_2_S signaling. Therefore, we need to further elucidate the molecular mechanisms by which H_2_S enhances seed germination and the growth and development of tobacco seedlings using metabolomics and transcriptomics approaches.

## Conclusions

In this study, we determined the effect of pre-soaking and irrigating roots with different NaHS concentrations on the growth and development of tobacco. Our results revealed that using exogenous NaHS could increase the endogenous H_2_S content by increasing *L-D/CD* expression and L-D/CD enzyme activity. However, the contents of endogenous H_2_S in tobacco are retained only for a brief time. Meanwhile, H_2_S could increase the speed of germination, seed vigor, root activity, the contents of photosynthetic pigments, and other agronomic traits. Additionally, different NaHS concentrations demonstrated no significant effect on the percentage of seed germination and carotene content. Moreover, 6.0 mM exogenous NaHS could significantly improve the SOD and POD enzymatic activities, thus implying that H_2_S could promote the growth and development of tobacco in the absence of abiotic stress and induce resistance to various oxidative stresses. In the future, a small-scale application of NaHS in tobacco cultivation will be conducted in the field experiment, and metabolomics and transcriptomics approaches will be used to further elucidate in-depth the molecular mechanisms of H_2_S in improving tobacco seedling growth and development.

## Materials and methods

### Tobacco materials and reagents

The tobacco (Yunyan 87) seeds were provided from Tobacco Research Institute of Hubei in Wuhan, China. NaHS was acquired from Aladdin Reagent Co., Ltd. (Shanghai, China) for use in this study. The different concentrations (0.0, 2.0, 4.0, 6.0, and 8.0 mM) of NaHS were used for pre-soaking of tobacco seeds and root-irrigation of tobacco seedlings. Tobacco seeds of uniform size and fullness were selected and disinfected with 3% sodium hypochlorite solution for 15 min, after which the filter paper containing the seeds must be opened and allowed to dry in the wind. It is imperative to execute this process on an ultra-sanitized surface to prevent any potential contamination. To promote seed germination, we ensured tobacco seeds remained hydrated with sterile water. Following a 24-h interval, we diligently sprayed all the boxes with sterile water. Subsequently, we positioned the boxes in an incubator, maintaining a constant temperature of 28 ± 2 °C and a relative humidity of 80 ± 5% throughout the germination test. The levels of NaHS were selected as described previously [54] with minor modifications and based on our previous study [31].

### Measuring the endogenous H_2_S levels

We determined the level of endogenous H_2_S as described previously [57]; however, the protocol was slightly modified. First, approximately 0.1 g of tobacco leaves or rhizomes were ground in 0.9 mL of 20 mmol/L Tris–HCl (pH 8.0) using a mortar and centrifuged. Next, the supernatant was collected in a flask, and 1% Zn (AC)_2_ was added to the flask and sealed using a rubber stopper. H_2_S released during the reaction was absorbed by Zn (AC)_2_. After the reaction at 37 °C for 40 min, we added 100 μL of 20 mmol/L DPD in 7.2 mol/L HCl to 100 μL of 30 mmol/L FeCl_3_ in 1.2 mol/L HCl and allowed it to react for 5 min. Finally, the absorbance values at 670 nm were measured, and we constructed a standard curve based on the Na_2_S concentration gradient. All experiments were performed thrice, and the final data were presented as the mean ± standard error of the three measurements.

### RNA extraction and quantitative Real-Time PCR (qRT-PCR)

Total RNA was extracted from tobacco tissues using RNAiso Plus reagent (TaKaRa, Shiga, Japan), purified using DNase I, and quantified using NanoDrop1 1000c spectrophotometer (Nanodrop Technologies, DE, USA). These RNA samples served as templates for synthesizing complementary DNA (cDNA) using the iScript cDNA Synthesis Kit (BioRad), following the instructions provided by the manufacturer. Next, *LCD(NW_015876407.1)* and *DCD(NW_015820577.1)* expression levels were measured on a BIO-RAD RT-PCR system (CFX96 C1000 Touch™ thermal cycler) using SsoFAST™ Eva Green Supermix (BIO-RAD, 1,725,201, CA, USA). *UBI3(NC_015438.3)* was used as an internal control. Finally, PCR products were sequenced to confirm the amplification of target genes. qRT-PCR was performed in 20 μl total volume with 1 μl of template cDNA from tenfold-diluted reverse transcription products, 10 μl SYBR, 1 μl each of upstream and downstream primers, and 7 μl ddH_2_O. The PCR thermal cycles were: 3 min at 95 °C for cDNA denaturation, followed by 40 cycles of 10 s at 95 °C, 20 s at 54 °C and 30 s at 72 °C. A final elongation step was performed for 10 min at 72 °C. Cycle threshold (C_t_) values for each gene of interest were averaged and normalized against the C_t_ value of the *UBI3* gene. The expression of each gene was determined from three replicates. The gene expression was then calibrated/normalized against the *UBI3* gene by using the 2^−ΔCt^ calculation: ΔCt = Ct_gene of interest_− Ct _UBI3*.*_ All experiments were performed thrice. All of the primer pairs used for qRT-PCR were checked for amplification specificity and are listed in Table 4.

**Table 4:** Primer sequences

Primers	Primers sequences (5’-3’)
*UBI3-F*	5’-GCCGACTACAACATCCAGAAGG-3’
*UBI3-R*	5’-TGCAACACAGCGAGCTTAACC-3’
*LCD-F*	5’-GGTTCGTCTGGCTGTGATTGATC-3’
*LCD-R*	5’-GGACCTCCTGGAATACAAGAAAGC-3’
*DCD-F*	5’-GTCCTGGGCCTCACACCTTAAT-3’
*DCD-R*	5’-CACGACAGTGATTGCTTTGGATGC-3’

### Determining seed germination

We performed germination tests for 14 days to evaluate the effect of NaHS on tobacco seed germination and the growth of seedlings. These samples (n = 5) were treated with 0.0, 2.0, 4.0, 6.0, and 8.0 mM NaHS. All experiments were performed in triplicates, and 50 tobacco seeds were used per replicate. Tobacco seeds of uniform size and fullness were selected and disinfected with 3% sodium hypochlorite solution for 15 min. Next, seeds were rinsed with sterile water and soaked in different concentrations of NaHS for 2 h. Finally, seeds were subjected to blotting using sterile filter paper, and 50 seeds were placed in each petri dish. All experiments were performed in triplicates. Seeds were germinated in a germination box containing two layers of filter paper. The moisture in the box required for seed germination was maintained using sterile water, and the boxes were placed in an incubator at 28 ± 2 ℃, where the relative humidity was 80 ± 5% throughout the germination test. We observed and recorded the number of germinated seeds daily. After 14 days of germination, the rate and index of germination and seed vigor index were calculated using the following formulae [58]:

$$\mathrm{Germination}\;\mathrm{rate}\:=\:(\mathrm{Number}\;\mathrm{of}\;\mathrm{germinated}\;\mathrm{seeds}/\mathrm{Total}\;\mathrm{number}\;\mathrm{of}\;\mathrm{seeds})\:\times\:100\%$$

$$\mathrm{Germination}\;\mathrm{index}\:=\:\Sigma\mathrm{Gt}/\mathrm{Dt}.$$

$$\mathrm{Seed}\;\mathrm{vigor}\;\mathrm{index}\:=\:\mathrm S\:\times\:(\Sigma\mathrm{Gt}/\mathrm{Dt}).$$

Where S is the mean dry weight, Gt is the number of germinated seeds in one day, and Dt is the number of germination days.

### Analyzing plant agronomic traits

When the size of the first and second main leaves was similar to the cotyledons and formed a cross shape, the tobacco seedlings were moved from the seedling tray to same-size pots, and the roots were treated with 10 mL of 0.0, 2.0, 4.0, 6.0, and 8.0 mM NaHS at an interval of 5 days thrice. Following 15 days of cultivation, the entire tobacco plants were harvested at the five-leaf period. The plant-relevant agronomic traits, including maximum width, length, and area of leaves, stem diameter, the height of plants, and the dry and wet weight of roots, were measured. The parameters of agronomic traits were measured using the YC/T142-2010 Tobacco agronomic traits survey and the measurement method. The root samples were dried at 70 ℃ for 72 h to maintain a constant weight, and the biomass of three samples was measured.

### Determining the content of photosynthetic pigments

The photosynthetic pigments in tobacco leaves were measured using the ethanol extraction method as previously described [59]. Briefly, fresh tobacco leaves from all treatment groups were collected, clipped (midvein removed), and mixed. Next, 0.2 g of freshly cut leaf tissue was weighed and incubated in 25 mL of 95% ethanol solution in a stoppered tube at 25 ℃ in the dark until the color changed to white (24 h) on a shaker for several hours. Next, the absorbance at 665 nm, 649 nm, and 470 nm was measured with the aid of an ultraviolet–visible spectrophotometer to determine the content of chlorophyll (Chl) *a*, Chl *b*, and carotenoid (Car *c*) in the supernatant. We used 95% ethanol as blank. The contents of photosynthetic pigments were calculated using the following formulas:

$$\mathrm{Ca}=13.70\;\times\;{\mathrm{OD}}_{665}-5.76\times{\mathrm{OD}}_{649}.$$

$$\mathrm{Cb}\;=25.80\;\times{\mathrm{OD}}_{649}-7.60\;\times{\mathrm{OD}}_{665}.$$

$$\mathrm{Cx}.\mathrm c=\left(1000\;\times\;{\mathrm{OD}}_{470}\;-2.05\;\times\mathrm{Ca}-114.80\;\times\mathrm{Cb}\right)/245.$$

$$\mathrm{Chl}\;\mathrm a=\mathrm{Ca}\;\times\;\mathrm V/\mathrm W\;\times\;1000.$$

$$\mathrm{Chl}\;\mathrm b\;=\mathrm V/\mathrm W\;\times1000.$$

$$\mathrm{Car}\;\mathrm c\;=\mathrm{Cx}.\mathrm c\;\times\;\mathrm V/\mathrm W\;\times\;1000.$$

where V is the extract volume, and W is the weight of the sample.

### Determination of the activities of H_2_S synthase L/D-CD

L/D-CD enzyme activity was measured by determining the amount of H_2_S released/minute by degrading L/D-Cys by L/D-CD enzymes as described previously [10]; [60] with minor modifications. First, we grounded 0.1 g of tobacco leaves or rhizomes with 0.9 mL of 20 mmol/L Tris–HCl (pH 8.0) in a mortar and centrifuged. Next, we added 100 μL of the supernatant to 100 μL of 0.8 mmol/L L/D-cysteine, 400 μL of 2.5 mmol/L DTT and 400 μL of 100 mmol/L of Tris–HCl. The pH value was adjusted to 9 for measuring the LCDs activity and 8 for measuring the DCDs activity. The content of H_2_S is presented in Sect. 1. After the reaction at 37 °C for 40 min, we added 00 μL of 20 mmol/L DPD in 7.2 mol/L HCl to 100 μL of 30 mmol/L FeCl_3_ in 1.2 mol/L HCl. Finally, we measured the absorbance at 670 nm after 5 min.

### Determination of root vigor

We measured root vigor using the 2,3,5-triphenyl tetrazolium chloride (TTC) method [61]. Briefly, we placed 0.5 g of root samples in 10 mL of equal volumes of phosphate buffer and 0.4% TTC in a tube, mixed, and incubated the setup at 37 °C for 2 h in the darkness in a temperature chamber until the root tip sections develop a red color. Then, we terminated the reaction using 2 mL of 1 mol/L sulfuric acid. Next, we cut the root tip with red color, completely immersed it in 10 mL methanol in a stoppered graduated test tube, and incubated it at a temperature of 30 ~ 40 °C until the root tip turned completely white. Finally, we measured the OD of the above extract at 485 nm using the spectrophotometric method (SP-756PC). The blank test was used as a reference. All experiments were performed in triplicates. The root vigor was calculated as follows:

$$\mathrm{Root}\;\mathrm{vigor}\;\mathrm{ACT}=\mathrm{CmW}\times\left[\upmu\text{g TTF/(g h)}\right].$$

where C is the amount of tetrazolium reduction (μg), W is the root weight (g), h is the time in h, and m is the dilution of extracted solution.

### Determining the POD activity

We determined the POD activity described previously [62]. Briefly, 5.0 g of washed tobacco leaves were treated with 0.0, 2.0, 4.0, 6.0, and 8.0 mM NaHS for 0, 5, 10, and 15 days, weighed, and homogenized separately using a mortar and pestle. The leaves were stored in 4 mL of 50 mM potassium phosphate buffer (pH 7.6) at 4 °C. We transferred the homogenate to a tube and centrifuged it at 3000 g for 10 min. Subsequently, we transferred the supernatant to a 25 mL volumetric flask. Next, we extracted the precipitate using 5 mL phosphate buffer twice and added the supernatant into the volumetric flask. The volume was fixed to the scale and stored at a low temperature. For the enzyme activity assay, we added 0.1 mL of the enzymatic extract, 2.9 mL in 0.05 mol/L phosphate buffer to 1.0 mL of 2% H_2_O_2_ and 0.05 mol/L guaiacol, and incubated it in a water bath at 37 ℃ for 15 min. The enzymatic extract was boiled for 5 min as the control. Next, the reaction system was transferred to an ice bath. The reaction was terminated using 2.0 mL of 20% trichloroacetic acid, filtered or centrifuged at a rate of 5000 g for 10 min, diluted, and the absorbance was measured at 470 nm.

The POD activity was calculated using the following formula:

The change value of A_470_ by 0.01 per minute was used as 1 unit of the POD activity (u).

$$\mathrm{POD}\;\mathrm{activity}=\frac{\triangle\text{A}470\times\text{Vt}}{\text{W}\times\text{Vs}\times0.01\times\text{t}}\lbrack\mathrm u\;\mathrm g-^1\;\min-^1\rbrack$$

where △A_470_ is the change value of absorbance during the reaction, W is the fresh weight of tobacco in g, t is the reaction time in minutes, Vt is the total volume of the enzymatic extract in mL, and Vs is the volume of the test enzymatic extract determined in mL.

### Determining the SOD activity

First, we treated 0.5 g of fresh tobacco leaves with 0.0, 2.0, 4.0, 6.0, and 8.0 mM NaHS for 0, 5, 10, and 15 days. Next, we homogenized and ground the leaves into a slurry using 5 mL of pre-chilled phosphate buffer in a pre-chilled mortar on an ice bath. Next, the homogenate was filtered using a cheesecloth and centrifuged at 1000 r/minutes for 20 min. Finally, the supernatant served as the crude enzyme extract. All the steps were performed at 0–4 °C. The SOD activity was measured as follows:

We added 0.05 mL of the enzymatic extract to 0.3 mL of 130 mmol/L methionine, 750 μmol/L nitroblue tetrazolium chloride (NBT), and 100 μmol/L EDTA-Na2 each, and 0.32 ml of 20 μmol/L riboflavin in a 0.05 mol/L potassium phosphate buffer (pH 7.8, Table 5. The reaction mixture was subjected to incubation for 20 min in a chamber under a 4000 lx fluorescent lamp. The reaction was initiated by turning on the fluorescent lamp and was terminated by turning the lamp off after 5 min. The increase in absorbance at 560 nm was used to measure blue formazan via NBT photoreduction. The reaction mixture without enzyme extract was used as a control and incubated in the darkness. One unit of SOD was the amount of enzyme required to suppress 50% of NBT photoreduction compared to the reaction mixture without the plant extract. We calculated the SOD activity as follows [63].

**Table 5:** Amount of each solution used for reaction

Reagent (enzyme)	Dosage (mL)	Final concentration
0.05 mol/L potassium phosphate buffer	1. 5	
130 mmol/L methionine	0.3	13 mmol/L
750 μmol /L NBT	0.3	75 μmol/L
100 μmol /L EDTA-Na2	0.3	10 μmol/L
20 μmol /L riboflavin	0. 32	0 μmol/L
the enzymatic extract	0.05	Two control tubes with buffer solution
Distilled H_2_O	0.25	
Total volume	3.0	

$$\mathrm{The total SOD}= \frac{({{\text{A}}}_{{\text{CK}}} -{{\text{A}}}_{{\text{E}}}) \times {\text{V}}}{0.5\times {{\text{A}}}_{{\text{CK}}}\times {\text{W}}\times {{\text{V}}}_{{\text{t}}}}$$

$$\mathrm{SOD}\;\mathrm{activity}\;=\mathrm{the}\;\mathrm{total}\;\mathrm{SOD}\;\mathrm{activity}/\mathrm{protein}\;\mathrm{content}.$$

ACK and AE are the absorbances of the illuminated control and sample tubes, respectively, V is the total volume of the sample tube in mL, Vt is the amount of test sample determined in mL, and W is the sample fresh weight in g.

### Statistical analysis

All experiments were performed thrice. The mean and standard error values are shown in the figures and tables. The bars with the different letters in lowercase and the same letters in lowercase indicate no significant difference. In addition, we performed a one-way analysis of variance and t-test to determine differences across multiple groups and between two groups, respectively.

## Data Availability

The data that support the results are included within the article or available from the corresponding author on reasonable request. *UBI3*, *LCD* and *DCD* genes sequences were downloaded from NCBI and the accession numbers were NC_015438.3(*UBI3*) NW_015876407.1(*LCD*) and NW_015820577.1(*DCD*), respectively.
